# Reduction of DUP in early intervention programmes: No pain… almost no gain

**DOI:** 10.1111/eip.13580

**Published:** 2024-05-28

**Authors:** Morgane Frischherz, Philippe Conus, Philippe Golay

**Affiliations:** ^1^ Faculty of Biology and Medicine University of Lausanne Lausanne Switzerland; ^2^ General Psychiatry Service, Treatment and Early Intervention in Psychosis Program (TIPP–Lausanne) Lausanne University Hospital and University of Lausanne Lausanne Switzerland; ^3^ Community Psychiatry Service, Department of Psychiatry Lausanne University Hospital and University of Lausanne Lausanne Switzerland; ^4^ Institute of Psychology, Faculty of Social and Political Science University of Lausanne Lausanne Switzerland

**Keywords:** duration of untreated psychosis, early intervention, first episode psychosis, psychosis, schizophrenia

## Abstract

**Aim:**

Considering the negative impact of long duration of untreated psychosis (DUP) on outcome, its reduction has become one of the aims of early intervention programmes. The TIPP programme (Treatment and early Intervention in Psychosis Program) was implemented in 2004 in Lausanne and hoped to reduce DUP, without any specific campaign in this regard, through the provision of accessible and specialized treatment. The aim of this study was to evaluate the evolution of patients' DUP over time and the characteristics of patients with extreme DUP.

**Methods:**

Clinical follow‐up data of 380 patients aged 18–35 years with a first psychotic episode who entered the TIPP programme between 2004 and 2017 were analysed. The evolution of DUP over time as well as referring entities and destination after the programme were assessed. The characteristics of patients with extreme DUPs (>percentile 90) were compared with that of other patients.

**Results:**

The mean value of the DUP was 452.11 days with a median of 88 days. DUP decreased only moderately over time. We also observe a decrease in discharges to specialized outpatient care at our university hospital. The main characteristics of patients with extreme DUP were early age of onset of psychosis, diagnosis of schizophrenia and presence of history of psychiatric treatment for other conditions before onset of psychosis.

**Conclusions:**

These figures suggest that the DUP has reduced over time but that without specific interventions at this level, this reduction is only moderate.

## 1 INTRODUCTION

Duration of untreated psychosis (DUP) is defined as the period between the onset of the first threshold psychotic episode and the initiation of specialized treatment. Long DUP has been shown to be associated with higher severity of symptoms during treatment and poorer outcome regarding symptoms remission, quality of life and functional level (Dama et al., 2019; Golay et al., 2016; Kane et al., 2016; Malla et al., 2014; Marshall et al., 2005; Schimmelmann et al., 2008; Souaiby et al., 2016). Although a specific cut‐off for ‘excessive’ DUP has not been defined, an American study showed that a DUP below 74 weeks was linked to better treatment response and higher quality of life after 3 years (Kane et al., 2016). However, much shorter DUP should be targeted. Indeed, in a previous paper (Golay et al., 2022), we showed that once DUP reached more than 3 weeks, the likelihood to reach a good level of functioning after 3 years decreased substantially.

Considering these elements, DUP is considered as a key marker of the performance of First Episode Psychosis (FEP) programmes in fidelity scales (Addington et al., 2018), and from a service use perspective, DUP is often used as a mean to monitor patients' access to care. Certain FEP programmes have incorporated community‐based campaigns to promote reduction of DUP through reduction of treatment delay. These campaigns have involved diverse media sources and community‐wide education strategies, which have proven temporarily successful with a reduction in DUP evident while the campaign is active, and an increase in DUP observed again once the campaign ends (Malla et al., 2005). In Lausanne, the TIPP programme (Treatment and early Intervention in Psychosis Program) was established in 2004. Due to lack of resources, a specific community‐based campaign could not be implemented. However, the programme still hoped to reduce DUP through the provision of accessible specialized treatment.

There are three goals in this study. First, we wanted to analyse the evolution of DUP over time in our programme and to see if, in the absence of any specific campaign, the mere implementation of the programme and its promotion over time through scientific publications and interactions with referring sources would have an impact on DUP. Second, we wanted to monitor the evolution of sources of referral and destination at the end of the 3 year programme over time. Finally, we wanted to establish the profile of patients with extreme DUP (>percentile 90) in order to identify patients who are at risk of a long DUP and to explore the evolution of the prevalence of such patients over time.

## 2 MATERIALS AND METHODS

### 2.1 Participants

The clinical follow‐up data of 380 patients aged 18–35 years with a first episode of psychosis who entered the TIPP programme between 2004 and 2017 were analysed. The TIPP is a specialized early psychosis programme attached to Lausanne University Hospital's Department of Psychiatry (Baumann et al., 2013). Patients eligible to the programme are aged 18–35, live in the hospital's catchment area (population about 350 000) and meet criteria for psychosis as defined by the ‘psychosis threshold’ subscale in the Comprehensive Assessment of At‐Risk Mental States (CAARMS) instrument (Yung et al., 2005). This psychotic disorder threshold is defined as frank psychotic symptoms such as delusions, hallucinations and thought disorder persisting for longer than 1 week and with a frequency of at least 3–6 times a week for longer than 1 h each time or daily for less than 1 h each time. This is a standard and widely used criteria for first episode psychosis threshold (Nelson et al., 2014).

Patients with psychosis related to intoxication or an organic brain disease, an IQ < 70, or who have been taking antipsychotic medication for more than 6 months are referred to other programmes. Patients attending the programme are referred to TIPP by various entities: general practitioners, families, private psychiatrists, psychiatric institutions, the Psychiatry Liaison Service and other Lausanne University Hospital departments (e.g., emergency, psychiatry). After a first contact by phone, the multidisciplinary team (including psychiatrists and case management nurses) verifies criteria before admitting patients. TIPP applies the principles of both case management interventions and assertive community treatment; patients are seen about 100 times over the 3‐year programme, primarily by their case manager but also by a resident physician or an intern in psychiatry. A consultant psychiatrist supervises each case.

Over 3 years, case managers are available to each patient up to twice a week. An Intensive Case Management team can provide additional support and treatment at any time during the treatment period. TIPP case managers remain involved, however, to ensure continuity of care. This study was carried out in accordance with the Declaration of Helsinki and was approved by the Human Research Ethics Committee of the Canton of Vaud (CER‐VD; protocol #2020‐00272). The data generated by the follow‐up of all patients were used in the study if the latter did not explicitly object to the use of their data for research purposes. All patients who received treatment in the programme within this timeframe could be included in this study.

### 2.2 Measures

All patients treated at TIPP are assessed at baseline, after 2 months, 6 months and then prospectively every 6 months, to monitor outcomes and adjust treatments. A specially designed questionnaire (the TIPP Initial Assessment Tool: TIAT, available upon request) is completed for all patients enrolled in the programme by case managers. It allows assessment of demographic characteristics, past medical history, exposure to life events as well as symptoms and functioning. It is completed based on information gathered from patients and their family over the first weeks of treatment and can be updated during follow up if new information emerges. Follow‐up assessments exploring various aspects of treatment and co‐morbidities as well as evolution of psychopathology and functional level are conducted by a psychologist and by case managers at baseline, after 2, 6, 12, 18, 24, 30 and 36 months in treatment. Symptoms assessments are conducted by a psychologist who is independent of patients' treatment and had received standardized training.

In order to be in line with most research in the domain, we defined DUP as the time between the onset of psychosis defined by the CAARMS instrument (Yung et al., 2005) and the admission to the TIPP. Here, psychotic disorder threshold is defined as having frank psychotic symptoms such as delusions, hallucinations and thought disorder persisting for longer than 1 week, with a frequency of at least 3–6 times a week for longer than 1 h each time or daily for less than 1 h each time. This is a standard and widely used criterion for first episode psychosis threshold (Nelson et al., 2014). The psychosis threshold and its time are determined prior admission based on an expert consensus between the TIPP psychiatrists and case managers using information from medical or hospitalization reports from treating psychiatrists if available, as well as from the detailed report of the clinician who addressed the patient to the program. If the psychosis threshold cannot be determined clearly based on these reports, further specialized clinical assessments are conducted based on the structured interview for psychosis‐risk syndromes (SIPS; McGlashan et al., 2001). Following this process, the clinical director of the TIPP completes the CAARMS.

To assess the patients' level of functioning during the programme, the Global Assessment of Functioning (GAF), the Social and Occupational Functional Assessment Scale (SOFAS) and the Premorbid Adjustment Scale (PAS) were used. The intensity of psychotic symptoms over time was measured using the Clinical Global Impression (CGI) scale, and the Positive and Negative Symptoms Scale (PANSS). DSM IV criteria were used to assess substance dependence.

### 2.3 Procedure

The relationship between time since programme implementation and DUP was first examined. To compare the socio‐demographic and psychiatric characteristics of the patients, two equal groups of 190 patients according to their date of entry into the programme were then created (first phase between 2004 and 2010; second phase between 2010 and 2017).

Patients with extreme DUPs (>90th percentile rank; ‘P90’) were identified in these two groups; then their profiles were compared with all patients with DUPs below P90 (‘reference group’). Three groups of patients were therefore formed: a reference group without extreme DUP with a date of entry into the programme between 2004 and 2017, a group with extreme DUP from 2004 to 2010 and a group with extreme DUP from 2010 to 2017.

Finally, to compare the evolution of the entry and exit points of patients between the beginning of the programme and today, we also compared the first quarter of patients who joined the programme at the beginning (90 patients) with a second group comprising the last quarter of patients who joined the programme (90 patients).

### 2.4 Statistical analysis

To assess the relationship between DUP and time since programme implementation, which are both continuous variables, we used the Spearman's Rho correlation coefficient. DUP and the delay since the programme beginning were expressed in days. We also performed a Mann–Whitney *U* test contrasting the two time periods. To compare the socio‐demographic and psychiatric characteristics of the patients, we used a Bayesian model comparison approach of Gaussian and multinomial models, which is an elegant alternative to the classical problem of multiple comparisons (Golay et al., 2019; Noël, 2020). Three groups were compared with each other: group 1 (reference, all patients with DUP < P90), group 2 (DUP > P90 from 2004 to 2010) and group 3 (DUP > P90 from 2010 to 2017). Within this model comparison framework, the homogeneous model (1, 2, 3) stated that there was no difference between the three groups. This model was compared to 4 other possible models: the heterogeneous model (1) (2) (3), which postulated that each group was different from the others and three ‘intermediate’ models (1, 2) (3), (1) (2, 3) and (1, 3) (2), which indicated that the data were derived from two different distributions with two similar groups and one group differing from the other two. The most likely model was determined based on the BIC coefficient, respectively, based on the exact likelihood depending on if it was a Gaussian or a multinomial model. To compare the proportions of the different entry and exit points between the groups, we also proceeded by model comparison. In this configuration, we analysed the contingency tables of entry and exit points between the three groups and between the first and last quarter of patients who joined the programme.

## 3 RESULTS

### 3.1 Evolution of DUP

The average DUP for the total sample was 452 days (SD = 872), while the median was 88 days (IQR = 461). Between 2004 and 2010, the average DUP was 514 days (SD = 926) and the median was 127 days (IQR = 586). Between 2011 and 2017, the mean DUP was 390 days (SD = 812) (24.1% decrease) and the median was 67 days (IQR = 332) (47.2% decrease).

The DUP considered as extreme (>P90) was greater than 1337 days. In the reference group (*N* = 342), the mean DUP was 217 days and the median 67 days. The mean DUP of the first extreme group was 2741 (median 1956 days) and 2463 days (median 2328 days) for the second extreme group. There were 24 patients with extreme DUP in group 2 (2004–2010) compared with only 14 patients with extreme DUP in group 3 (2010–2017). Regarding the evolution of the DUP over time, a decrease in its duration was observed (Spearman's Rho correlation coefficient: −0.124, *p* = .016). However, the Mann–Whitney *U* test contrasting both time periods (2004–2010 vs. 2011–2017) was not significant (*U* = 16654.00, *p* = .192).

### 3.2 Referrals

Regarding the sources of referral to the programme, there was no difference between the two groups (first phase between 2004 and 2010; second phase between 2010 and 2017; Table 1). There were also no differences regarding source of referral between reference group and extreme groups (Table 2). The majority of patients entered the TIPP programme through the psychiatric hospital and were referred to a specialized outpatient units at Lausanne University Hospital (CHUV) at the end of the programme.

**TABLE 1:** Comparison of programme entry and exit points over time according to the start date of the programme.

	Group 1 (2004–2010) *N* = 190	Group 2 (2010–2017) *N* = 190	Best model^a^	Bayes factor against null hypothesis^b^	Probability of the model to be true^c^
Entry in TIPP, % (*n*)			(1, 2)	1.0000	0.6509
Psychiatric hospital	64.8 (118.0)	64.9 (111.0)			
Assertive community treatment	8.2 (15.0)	3.5 (6.0)			
Child and adolescent psychiatry	0.5 (1.0)	2.3 (4.0)			
External psychiatrist	6.6 (12.0)	4.1 (7.0)			
Emergency services	12.1 (22.0)	8.8 (15.0)			
Ambulatory consultation	7.1 (13.0)	8.2 (14.0)			
Other	0.5 (1.0)	8.2 (14.0)			
Exit from TIPP, % (*n*)			(1, 2)	1.0000	0.9781
No follow‐up required	2.8 (4.0)	3.6 (4.0)			
Specialized outpatient follow‐up at CHUV	49.3 (71.0)	37.3 (41.0)			
General outpatient follow‐up at CHUV	14.6 (21.0)	14.5 (16.0)			
General outpatient follow‐up external	20.8 (30.0)	35.5 (39.0)			
Follow‐up by general practitioner	6.3 (9.0)	5.5 (6.0)			
Other	6.3 (9.0)	3.6 (4.0)			

Abbreviation: TIPP, Treatment and early Intervention in Psychosis Program.

^a^ Based on the exact likelihood.

^b^ Bayes factor comparing the best model to the homogeneous model (1, 2).

^c^ Relative to all possible models ((1, 2)/(1) (2)).

**TABLE 2:** Comparison of entry and exit points for patients with extreme DUP and other patients.

	Group 1 *N* = 342	Extreme DUP		Best model^a^	Bayes factor against null hypothesis^b^	Probability of the model to be true^c^
		Group 2 before 01 January 2010 *N* = 24	Group 3 after 01 January 2010 *N* = 14			
Entry in TIPP, % (*n*)				(1, 2, 3)	1.0000	0.8091
Psychiatric hospital	65.8 (210.0)	50.0 (11.0)	66.7 (8.0)			
Assertive community treatment	6.3 (20.0)	4.5 (1.0)	0 (0)			
Child and adolescent psychiatry	1.3 (4.0)	0.0 (0.0)	8.3 (1.0)			
External psychiatrist	5.0 (16.0)	13.6 (3.0)	0.0 (0.0)			
Emergency services	10.7 (34.0)	13.6 (3.0)	0.0 (0.0)			
Ambulatory consultation	6.9 (22.0)	18.2 (4.0)	8.3 (1.0)			
Other	4.1 (13.0)	0.0 (0.0)	16.7 (2.0)			
Exit from TIPP, % (*n*)				(1, 2, 3)	1.0000	0.8890
No follow‐up required	3.5 (8.0)	0.0 (0.0)	0.0 (0.0)			
Specialized outpatient follow‐up at CHUV	43.0 (99.0)	71.4 (10.0)	30.0 (3.0)			
General outpatient follow‐up at CHUV	14.3 (33.0)	7.1 (1.0)	30.0 (3.0)			
General outpatient follow‐up external	27.0 (62.0)	21.4 (3.0)	40.0 (4.0)			
Follow‐up by general practitioner	6.5 (15.0)	0.0 (0.0)	0.0 (0.0)			
Other	5.7 (13.0)	0.0 (0.0)	0.0 (0.0)			

Abbreviations: DUP, duration of untreated psychosis; TIPP, Treatment and early Intervention in Psychosis Program.

^a^ Based on exact likelihood.

^b^ Bayes factor comparing the best model to the homogeneous model (1, 2, 3).

^c^ Compared to all possible models ((1, 2, 3)/(1, 2) (3)/(1) (2, 3)/(1, 3) (2)/(1) (2) (3)).

There was, however, a difference when comparing the sources of referral and destination after treatment between the first and last quarter of patients to have joined the programme, regardless of their DUP (Table 3). Results showed there was an increase in ‘other’ sources of referral (e.g., general practitioners, training institutes or schools) after end of TIPP treatment. Results also showed a decrease in referral to specialized outpatient units at the CHUV and an increase in referral to private psychiatrists after TIPP treatment.

**TABLE 3:** Comparison of entry and exit points between the first (25%) and last (25%) patients to join the programme.

	(1) First quarter of patients *N* = 90	(2) Last quarter of patients *N* = 90	Best model^a^	Bayes factor against null hypothesis^b^	Probability of the model to be true^c^
Entry in TIPP, % (*n*)			(1) (2)	5.5541	0.8474
Psychiatric hospital	56.4 (53.0)	59.0 (49.0)			
Assertive community treatment	13.8 (13.0)	3.6 (3.0)			
Child and adolescent psychiatry	0.0 (0.0)	1.2 (1.0)			
External psychiatrist	6.4 (6.0)	1.2 (1.0)			
Emergency services	13.8 (13.0)	8.4 (7.0)			
Ambulatory consultation	8.5 (8.0)	13.3 (11.0)			
Other	1.1 (1.0)	13.3 (11.0)			
Exit from TIPP, % (*n*)			(1) (2)	1.2587	0.5573
No follow‐up required	1.3 (1.0)	0.0 (0.0)			
Specialized outpatient follow‐up at CHUV	50.6 (39.0)	23.3 (10.0)			
General outpatient follow‐up at CHUV	16.9 (15.0)	18.6 (8.0)			
General outpatient follow‐up external	19.5 (15.0)	46.5 (20.0)			
Follow‐up by general practitioner	3.9 (3.0)	7.0 (3.0)			
Other	7.8 (6.0)	4.7 (2.0)			

Abbreviation: TIPP, Treatment and early Intervention in Psychosis Program.

^a^ Based on the exact likelihood.

^b^ Bayes factor comparing the best model to the homogeneous model (1, 2).

^c^ Relative to all possible models ((1, 2)/(1) (2)).

### 3.3 Patients' profiles

Socio‐demographic and clinical profiles of the reference group and the two groups with extreme DUP >P90 were relatively similar with several exceptions (Tables 4 and 5): First, patients with extreme DUP had an earlier age of onset than the reference group. Second, presence of a history of previous psychiatric treatment was more frequent in the extreme DUP groups. Finally, there was more diagnoses of schizophrenia in the groups with extreme DUP.

**TABLE 4:** Comparison of social demographic characteristics of patients with extreme DUP and other patients.

	Group 1 *N* = 342	Extreme DUP		Best model^a^	Bayes factor against null hypothesis^b^	Probability of the model to be true^c^
		Group 2 before 01 January 2010 *N* = 24	Group 3 after 01 January 2010 *N* = 14			
Gender, % male (*n*)	63.700 (218.000)	70.800 (17.000)	78.600 (11.000)	(1, 2, 3)	1.0000	0.4213
Age of onset in years, *M* (SD)	23.670 (4.690)	18.500 (6.010)	18.640 (3.570)	(1), (2, 3)	9.59 × 10^6^	0.9505
Age at the beginning of the programme, in years *M* (SD)	24.420 (4.640)	25.170 (4.840)	26.360 (4.220)	(1, 2, 3)	1.0000	0.7183
Socio‐economical level, % (*N*) Low Intermediate High	20.500 (70.000) 40.900 (140.000) 38.6 00(132.000)	16.700 (4.000) 54.200 (13.000) 29.200 (7.000)	14.300 (2.000) 57.100 (8.000) 28.600 (4.000)	(1, 2, 3)	1.0000	0.5937
Education in years, *M* (SD)	9.990 (2.599)	9.900 (4.266)	11.540 (3.205)	(1, 2, 3)	1.0000	0.6270
Marital status, % (*N*)				(1, 2, 3)	1.0000	0.9368
Single	84.200 (283.000)	73.900 (17.000)	85.700 (12.000)			
Married	8.300 (28.000)	17.400 (4.000)	14.300 (2.000)			
Divorced	3.300 (11.000)	4.300 (1.000)	0.000 (0.000)			
Cohabitation	4.200 (14.000)	4.300 (1.000)	0 .000 (0.000)			
Professional activity, % (*n*)				(1, 3), (2)	14.9539	0.6475
Full time job	9.300 (31.000)	29.200 (7.000)	0.000 (0.000)			
Student/traineeship	19.500 (65.000)	8.300 (2.000)	0.000 (0.000)			
Part time job	3.000 (10.000)	4.200 (1.000)	0.000 (0.000)			
Disability annuity	1.800 (6.000)	12.500 (3.000)	7.100 (1.000)			
On sickness leave	20.100 (67.000)	8.300 (2.000)	35.700 (5.000)			
Unemployed	46.400 (155.000)	37.500 (9.000)	57.100 (8.000)			
Lifestyle, % (*n*)				(1, 2, 3)	1.0000	0.5617
Independent household	23.600 (78.000)	21.700 (5.000)	7.100 (1.000)			
With friends	22.400 (74.000)	39.100 (9.000)	7.100 (1.000)			
Family	43.300 (143.000)	39.100 (9.000)	71.400 (10.000)			
Pension/care home	4.500 (15.000)	0.000 (0.000)	0.000 (0.000)			
Unsettled (hostel, shelter, homeless)	6.100 (20.000)	0.000 (0.000)	14.300 (2.000)			
Trauma, % (*n*)	31.300 (105.000)	33.300 (8.000)	50.000 (7.000)	(1, 2, 3)	1.0000	0.3729
Migration in adversity, % (*n*)	31.800 (98.000)	17.400 (4.000)	18.200 (2.000)	(1, 2, 3)	1.0000	0.3149
Forensic history % (*n*)	13.400 (39.000)	18.200 (4.000)	0.000 (0.000)	(1, 2, 3)	1.0000	0.5066
Offences during programme % (*n*)	10.900 (22.000)	33.300 (2.000)	7.700 (1.000)	(1,3) (2)	1.2320	0.4165

Abbreviation: DUP, duration of untreated psychosis.

^a^ Based on the BIC coefficient or the exact likelihood.

^b^ Bayes factor comparing the best model to the homogeneous model (1, 2, 3).

^c^ Compared to all possible models ((1, 2, 3)/(1, 2) (3)/(1) (2, 3)/(1, 3) (2)/(1) (2) (3)).

**TABLE 5:** Comparison of clinical characteristics of patients with extreme DUP and other patients.

	Group 1 *N* = 342	Extreme DUP		Best model^a^	Bayes factor against null hypothesis^b^	Probability of the model to be true^c^
		Group 2 before 01 January 2010 *N* = 24	Group 3 after 01 January 2010 *N* = 14			
Premorbid adjustment (PAS), *M* (SD)						
PAS childhood	0.300 (0.170)	0.360 (0.280)	0.200 (0.180)	(1, 2, 3)	1.0000	0.6009
PAS early adolescence	0.310 (0.170)	0.400 (0.250)	0.240 (0.210)	(1, 3) (2)	1.0921	0.4276
PAS social	0.280 (0.200)	0.380 (0.300)	0.200 (0.230)	(1, 2, 3)	1.0000	0.4977
PAS academic	0.340 (0.200)	0.410 (0.240)	0.240 (0.180)	(1, 2, 3)	1.0000	0.6278
PAS total	0.300 (0.160)	0.380 (0.260)	0.210 (0.180)	(1, 2, 3)	1.0000	0.4834
Past suicide attempt, % (*n*)	13.200 (43.000)	31.800 (7.000)	21.400 (3.000)	(1,3), (2)	2.2521	0.3538
Psychiatric history % (*n*)	57.700 (192.000)	79.200 (19.000)	78.600 (11.000)	(1) (2,3)	5.6077	0.5032
Familial psychiatric history % (*n*)	55.200 (171.000)	77.300 (17.000)	64.300 (9.000)	(1, 3) (2)	1.8874	0.3473
Familial schizophrenia history, % (*n*)	21.700 (59.000)	26.300 (5.000)	10.000 (1.000)	(1, 2, 3)	1.0000	0.5046
Lifetime alcohol use, % (*n*)						
Abuse	22.100 (73.000)	23.800 (5.000)	14.300 (2.000)	(1, 2, 3)	1.0000	0.5550
Dependence	7.300 (24.000)	9.500 (2.000)	7.100 (1.000)	(1, 2, 3)	1.0000	0.6608
Lifetime cannabis use % (*n*)						
Abuse	35.000 (115.000)	36.400 (8.000)	21.400 (3.000)	(1, 2, 3)	1.0000	0.4743
Dependence	27.700 (91.000)	36.400 (8.000)	14.300 (2.000)	(1, 2, 3)	1.0000	0.4437
Lifetime other substance use, % (*n*)						
Abuse	10.700 (36.000)	21.700 (5.000)	14.300 (2.000)	(1, 2, 3)	1.0000	0.4116
Dependence	5.300 (18.000)	8.700 (2.000)	14.300 (2.000)	(1, 2, 3)	1.0000	0.5052
Insight at presentation				(1,2) (3)	1.1926	0.4182
Absent	31.300 (103.000)	45.500 (10.000)	7.100 (1.000)			
Partial	46.500 (153.000)	40.900 (9.000)	64.300 (9.000)			
Complete	22.200 (73.000)	13.600 (3.000)	28.600 (4.000)			
GAF programme entry, *M* (SD)	42.070 (17.075)	31.740 (15.289)	50.580 (18.520)	(1,3) (2)	3.4423	0.6099
GAF worst during psychosis, *M* (SD)	28.130 (11.154)	25.900 (13.285)	35.710 (7.600)	(1,2) (3)	1.3176	0.5087
SOFAS programme entry, *M* (SD)	43.120 (16.072)	36.330 (15.423)	53.170 (15.654)	(1, 2, 3)	1.0000	0.4105
SOFAS worst during psychosis, *M* (SD)	30.210 (11.253)	29.760 (14.570)	36.710 (5.823)	(1, 2, 3)	1.0000	0.5907
CGI programme entry, *M* (SD)	4.570 (1.419)	5.050 (1.146)	3.920 (1.084)	(1, 2, 3)	1.0000	0.6617
CGI higher during psychosis, *M* (SD)	5.740 (0.768)	5.800 (0.894)	5.330 (0.880)	(1, 2, 3)	1.0000	0.6927
Diagnostic, % (*n*)				(1), (2, 3)	6.1842	0.7318
Schizophrenia	55.000 (188.000)	91.700 (22.000)	78.600 (11.000)			
Schizophreniform/brief	13.700 (47.000)	0.000 (0.000)	0.000 (0.000)			
Schizo‐affective	9.100 (31.000)	8.300 (2.000)	7.100 (1.000)			
Major depressions	4.700 (16.000)	0.000 (0.000)	0.000 (0.000)			
Bipolar disorder	7.300 (25.000)	0.000 (0.000)	0.000 (0.000)			
Other	10.200 (35.000)	0.000 (0.000)	14.300 (2.000)			

Abbreviations: CGI, Clinical Global Impression; GAF, Global Assessment of Functioning; PAS, Premorbid Adjustment Scale; SOFAS, Social and Occupational Functional Assessment Scale.

^a^ Based on the BIC coefficient or the exact likelihood.

^b^ Bayes factor comparing the best model to the homogeneous model (1, 2, 3).

^c^ Compared to all possible models ((1, 2, 3)/(1, 2) (3)/(1) (2, 3)/(1, 3) (2)/(1) (2) (3)).

Group 2 (extreme DUP from 2004 to 2010) also differed in several ways from the reference group and from group 3. First, there was a higher proportion of full‐time work in group 2. Second, group 2 had poorer premorbid adjustment scores in early adolescence and a lower average GAF score than the other two groups. Group 2 also had a higher proportion of suicide attempts, more frequent family psychiatric history and a higher prevalence of legal offences committed during the TIPP programme.

Group 3 (extreme DUP from 2010 to 2017) also differed in two aspects from the other two groups: patients of this group had better insight at entry and a lower GAF score during psychosis.

## 4 DISCUSSION

There are three main findings stemming from our research. First, while DUP decreased over time, it did so only moderately and not in all analysis, and DUP remained significantly above recommendations. Second, the number of patients entering TIPP with extreme DUP decreased over time and the characteristics of these patients evolved over time. Third, we may hypothesize that the number of patients who need intense care after TIPP treatment, such as treatment delivered by the other specialized outpatient units at our department of psychiatry, diminished over time.

Globally, our results suggest that DUP has decreased over time, despite the absence of any large‐scale strategy in the general population in this regard. This may be partly linked to a decrease in the number of new patients with extreme DUP, but to a limited extend, considering the median DUP, marginally impacted by extreme values, dropped by 47%. It is important at this point to distinguish two components of DUP, which are the ‘help seeking delay’ and the ‘treatment delay’. The implementation of specialized early intervention programmes, the availability of assertive case management and the promotion of such strategies among the usual partners of our service likely had an impact on treatment delay. This hypothesis is supported by the observation that the number of patients entering TIPP via a hospitalization diminished over time, suggesting both that other partners had identified the programme and that patients' mental state was less deteriorated when entering TIPP. However, a median DUP of 67 days remains three times longer than current recommendations (8) and suggests more needs to be done. A meta‐analysis (Albert & Weibell, 2019) of different early intervention programmes around the world has shown that reduction of DUP remains challenging.

The community wide education strategies can probably reduce the help seeking delay more than the treatment delay, as this education would also need to be directed towards health professionals. A study conducted recently in Norway (Ferrara et al., 2019) showed that the reduction in DUP that could be observed after a large public campaign was short‐lived once the campaign was over. It is therefore likely that due to stigma and other factors, a deeper‐rooted strategy must be implemented and be directed towards both the public and health professionals. The Headspace approach in Australia may contribute to this by offering generic entry points for young people where access to mental health care is embedded within a larger focus on general health.

Patients with extreme DUP also had similar exit points from the programme as reference, which suggest they did not have an increased need for specialist follow‐up. This is a hypothesis as we cannot exclude that there could be other factors causing the reduction in referrals to specialized outpatient units, other than purely patient need. This results also need careful examination because, on the one hand, it may suggest that DUP did not have a long‐lasting effect (which is in contradiction with the whole concept of DUP reduction). On the other hand, it may suggest that the early intervention programme was able to take care of those patients adequately and was successful in allowing them to only need a standard and not reinforced follow‐up after 3 years of treatment.

Furthermore, the profile of the group of patients with extreme DUP between 2010 and 2017 (group 3) was more similar with the reference group than group 2 (DUP > P90 between 2004 and 2010). This suggests a decrease in comorbidities at programme entry with more recent patients. A study showed that after an early intervention programme was promoted, patients who had been ill for long periods of time and had a higher level of psychopathology were brought into treatment (Malla et al., 2005). We could hypothesize that patients with such unmet needs were much rarer after several years of programme availability. The higher proportion of full‐time work in group 2 was unexpected, given this group has a higher mean DUP and a higher number of extreme DUPs compared with group 3. This finding is difficult to explain and, if replicated in other samples, should be further studied.

There was, however, a difference when comparing the sources of referral after treatment between the first and last quarter of patients to have joined the programme, regardless of their DUP. The increased variety of ‘other’ sources of referral after the TIPP treatment may suggest increased awareness and visibility of the programme.

Extreme DUP was strongly correlated with early age of onset, psychiatric history and diagnosis of schizophrenia. These results are consistent with those of other studies (Apeldoorn et al., 2014; Ballageer et al., 2005; Schimmelmann et al., 2007; Schimmelmann et al., 2008). There is obviously a difficulty in identifying early psychosis in younger people. This may be due to a misattribution of symptoms to problems associated with adolescence and less obvious symptoms. Two studies (Ballageer et al., 2005; Schimmelmann et al., 2007) have indeed shown that psychosis beginning in adolescence has more negative symptoms than adult psychosis. These negative symptoms can mask positive symptoms and delay diagnosis. Finally, it has also been shown that a more insidious mode of onset of psychosis with fewer effects on patient functioning was also correlated with a longer DUP (Morgan et al., 2006; Schimmelmann et al., 2008).

This study has some limitations. First, for a better understanding of the course of these extreme DUPs, a detailed analysis of each patient's records would be necessary, and this information was not included in the present study. Second, our DUP measure was based on an expert consensus for the psychosis threshold between the TIPP clinicians rather than on tools specifically designed to measure DUP. The measurement of DUP could also be dependent of memory of past events and assessing the precise moment of onset becomes imprecise as time goes by.

## 5 CONCLUSION

Overall, the programme was successful in moderately reducing DUP and promoting access to care. One of the main risk factors for long DUP was the early age of onset of psychosis. It would therefore be interesting to strengthen prevention and awareness programmes for younger people. To achieve a greater change in the DUP and reach the ideal threshold of 3 weeks, there is a need for stronger mental health promotion with long‐term public information campaigns in Switzerland. The stigmatization of psychiatric patients also remains an obstacle to access to care that relies on society and cannot be changed by a single programme.

## FUNDING INFORMATION

This study was based on institutional funding.

## CONFLICT OF INTEREST STATEMENT

The authors declare that they have no competing interests.

## Data Availability

The data that support the findings of this study are available on request from the corresponding author. The data are not publicly available due to privacy or ethical restrictions.
